# Public Health in Rajasthan

**Published:** 2008-04

**Authors:** Arun Kumar Aggarwal

**Affiliations:** Dr. Arun Kumar Aggarwal School of Public Health, PGIMER, Chandigarh - 160 012, India. E-mail: aggak63@rediffmail.com

The book *Public Health in Rajasthan*, published in 2007 has 227 pages, divided into 25 chapters and 5 Appendices. Book has attractive title page and is printed on good quality paper with a comfortable font size. Chapters cover wide range of subjects related to National Rural Health Mission, Reproductive and Child Health, Integrated Child Development Services, National programs on Tuberculosis, Leprosy, HIV/AIDS, Blindness, National Vector Borne Disease Control Program, Integrated Disease Surveillance Project, Computerization and Management Information System, Medicolegal aspects, Consumer Protection Act, and Behavior Change Communication for Health, etc. There are two chapters devoted to role of The United Nations Children's Fund (UNICEF) and The United Nations Fund for Population Activities (UNFPA) in Rajasthan. Brief description of the projects undertaken through these agencies is useful. Galaxy of 21 prominent experts have contributed in this book. Each chapter provides a brief description of the state-specific program and has been written to act like an operational guide for the implementation of the program. Authors have nicely and succinctly compiled the information available at various places, and have also given resource references for further reading at the end of each chapter. Editor has very rightly claimed that the book can be labeled as a rapid reader for the doctors working at peripheral facility. This can also be good resource for public health students undertaking Masters of public health (MPH) or Doctorate in Medicine (MD) in the specialty. This would also be a good resource for all States to enable them to draft their own State-specific resource books and to have a ready reference for the programs that are operational in Rajasthan.

## About the Editor

Prof. Shiv Chander Mathur, The Editor of the book is a prominent public health expert with national and international teaching training and research experience. He has published more than 50 papers and two monographs on public health. He has also received Government of India merit award for his writings on primary health care. For any further queries readers may contact the editor. E-mail: shiv_mathur@hotmail.com.

